# A positive feedback loop between Gli1 and tyrosine kinase Hck amplifies shh signaling activities in medulloblastoma

**DOI:** 10.1038/oncsis.2015.38

**Published:** 2015-11-30

**Authors:** X Shi, X Zhan, J Wu

**Affiliations:** 1 Department of Physiology, University of Texas Southwestern Medical Center, Dallas, TX, USA

## Abstract

Sonic hedgehog (Shh) signaling is critical during normal development, and the abnormal activation of the Shh pathway is involved in many human cancers. As a target gene of the Shh pathway and as a transcription activator downstream of Shh signaling, Gli1 autoregulates and increases Shh signaling output. Gli1 is one of the key oncogenic factors in Shh-induced tumors such as medulloblastoma. Gli1 is posttranslationally modified, but the nature of the active form of Gli1 was unclear. Here we identified a Src family kinase Hck as a novel activator of Gli1. In Shh-responsive NIH3T3 cells, Hck interacts with Gli1 and phosphorylates multiple tyrosine residues in Gli1. Gli1-mediated target gene activation was significantly enhanced by Hck with both kinase activity-dependent and -independent mechanisms. We provide evidence showing that Hck disrupts the interaction between Gli1 and its inhibitor Sufu. In both NIH3T3 cells and cerebellum granule neuron precursors, the *Hck* gene is also a direct target of Gli1. Therefore, Gli1 and Hck form a positive feedback loop that amplifies Shh signaling transcription outcomes. In Shh-induced medulloblastoma, Hck is highly expressed and Gli1 is tyrosine phosphorylated, which may enhance the tumorigenic effects of the *Gli1* oncogene. RNAi-mediated inhibition of *Hck* expression significantly repressed medulloblastoma cell growth. In summary, a novel positive feedback loop contributes to maximal Gli1 oncogenic activities in Shh-induced tumors such as medulloblastoma.

## Introduction

Sonic hedgehog (Shh) signaling has critical roles in many development processes, and dysregulation of Shh signaling has been implicated in diseases and cancers such as those in cerebellum, skin, pancreas, prostate and lung.^1, 2, 3, 4, 5, 6^ In cerebellum during early postnatal development, Shh secreted from Purkinje neurons functions as a mitogen to stimulate the proliferation of cerebellum granular neuron precursor (CGNP) cells.^7, 8, 9, 10^ Mutations leading to constitutively active Shh signaling in CGNPs cause CGNP over-proliferation and Shh-type medulloblastoma, which accounts for 25% of all medulloblastoma cases and is the most frequent malignant childhood brain tumor.^11, 12, 13, 14, 15^ Shh signaling transduced by Patched (Ptch1) and Smoothened (Smo) induces target gene expression by activating Gli transcription activators.^1, 3, 16, 17^ Gli1 is a sensitive Shh target gene and functions solely as a transcription activator in response to Shh signaling. Thus it forms an auto-positive feedback loop that enhances Shh signaling outcomes.^5, 18^ Although Gli1 is not essential for development, it is a potent oncogene and is required for Shh-induced tumor growth.^19, 20, 21^ Gli1 expression is elevated in many cancer types with elevated Shh signaling.^3^ Inhibiting Gli1 activity would likely be an effective approach for treating these cancers. Thus, understanding the largely unknown mechanisms of Gli1 activation will provide insights into the mechanism of cancer growth and will guide development of treatments.^22, 23^

An important regulator of Gli1 activities is the inhibitor Sufu, which sequesters Gli1 in the cytoplasm and also inhibits Gli1 activities in the nucleus.^22, 24, 25, 26^ In addition, Gli1 activities are regulated by posttranslational modification events such as Ser/Thr phosphorylation. It can also be acetylated, ubiquitinated and sumoylated.^6, 26, 27, 28^ Several posttranslational modifications potentially interrupt the Gli1–Sufu interactions and release Gli1 from the inhibition by Sufu.^22, 26^ Gli1 modification enzymes such as histone deacetylases and atypical protein kinase C (aPKC) family members ι and λ are promising targets for the treatment of Shh-related cancers.^28, 29^

Several Tyr residues in Gli1 are conserved, but until our study, it was not known whether Gli1 was Tyr phosphorylated or whether tyrosine kinases function in regulating Gli1 activities. In mammals, there are 10 families of nonreceptor tyrosine kinases.^30, 31^ The Src family, consisting of Src, Hck, Lyn, Fyn, Fgr, Blk, Lck, Yes and Ylk, play essential roles in malignant transformation and tumor progression.^32, 33^ Besides the kinase activities, Src family kinases also display kinase activity-independent functions,^34, 35^ mostly through protein-protein interactions. The Src family kinase Hck has a known function in hematopoiesis.^36, 37^ Interestingly, *Hck* was identified in a genome-wide study of potential Gli1 binding genes in CGNPs and in Shh-type medulloblastoma.^38^ As Shh/Gli target genes such as *Gli1*, *Ptch1*, *Hhip* and *aPKC-ι/λ* are Shh pathway regulators, it is possible that Hck also regulates Shh signaling.

In this report, we show that *Hck* is a direct target gene of Shh signaling and can be activated by Gli1 in both NIH3T3 cells and in CGNPs. Hck interacts with Gli1 and phosphorylates it at multiple Tyr residues. Hck enhances Gli1-mediated target gene activation through both kinase activity-dependent and -independent mechanisms. We provide evidences showing that Hck releases Gli1 from Sufu inhibition by disrupting the Gli1–Sufu interaction. Therefore, Gli1 and Hck form a positive feedback loop to amplify Shh signaling outcomes. In Shh-induced medulloblastoma, both Gli1 and Hck are expressed at high levels and Gli1 is Tyr phosphorylated. RNAi-mediated inhibition of *Hck* expression significantly inhibited tumor cell growth. Thus, Gli1–Hck positive feedback loop enhances Gli1 oncogenic effects and contribute to the growth of medulloblastoma.

## Results

### *Hck* is a direct target of Gli1 in response to Shh signaling

**Figure 1:**
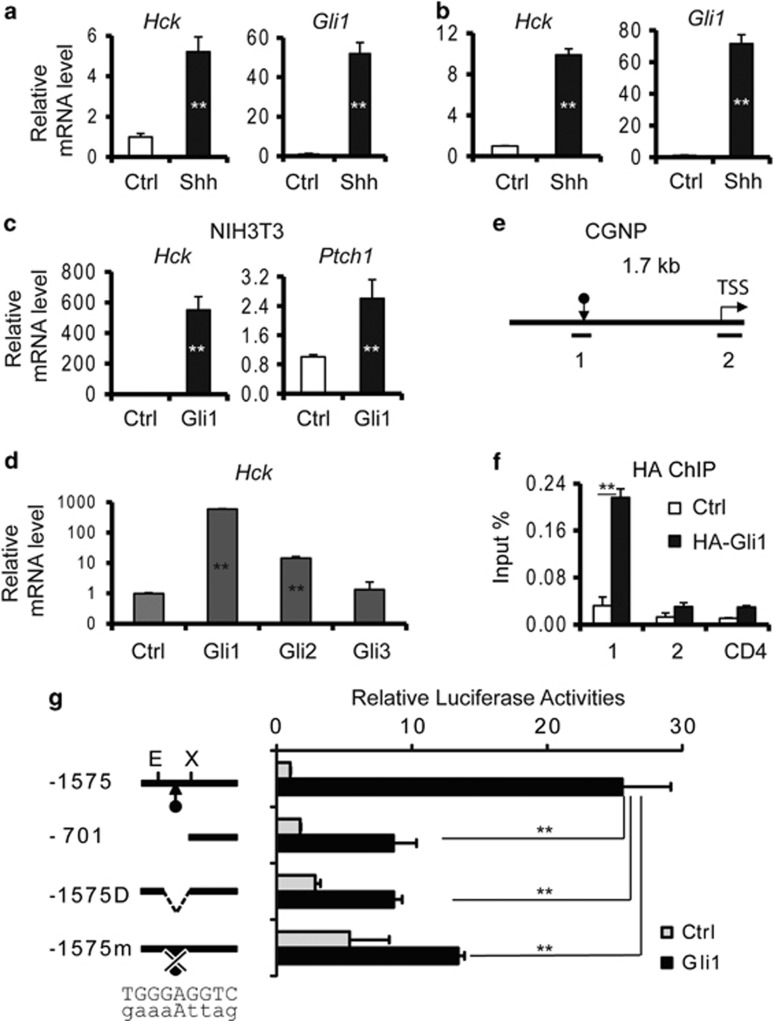
*Hck* is a direct Shh/Gli1 target gene. (**a**) NIH3T3 cells were treated with or without Shh-conditioned media. Levels of *Hck* and *Gli1* mRNAs were determined by RT–qPCR. (**b**) *Hck* and *Gli1* mRNA levels were determined by RT–qPCR in CGNP cells treated with or without Shh-conditioned media. (**c**) NIH3T3 cells were infected with empty vector or lentiviruses for expression of Gli1 proteins. Levels of *Hck* and Shh target gene *Ptch1* were determined. (**d**) Levels of *Hck* were determined by RT–qPCR in NIH3T3 cells infected with lentiviruses expressing different Gli proteins. (**e**) A schematic map of the 1.7 kb *Hck* regulatory region used for ChIP and reporter assays. The Gli binding site (indicated by the arrow) and the transcription start site (TSS) are shown. Small bars indicate the location of ChIP PCR products. (**f**) NIH3T3 cells were infected with empty vector control or HA-Gli1 expressing lentiviruses. ChIP-qPCR analyses were performed with anti-HA antibody. A region in *CD4* was used as a negative control. (**g**) The 1.7 kb wild-type full-length *Hck* enhancer region (−1575; E: EcoRI site. X: XhoI site) and mutants including a truncated construct (−701), a construct with the putative Gli1 binding site deleted (−1575D) or mutated (−1575 m) were cloned upstream of a luciferase reporter gene. Plasmids expressing control (empty vector) or Gli1 proteins were co-transfected with reporters into NIH3T3 cells. Relative luciferase activities are shown on the right. Presented are means plus s.d. Statistical analyses were performed using the Student's *t*-test; **P<0.01. qPCR, quantitative PCR.

In an experiment using RNA-seq designed to identify Shh-responsive genes in mouse embryonic fibroblasts (MEFs), we observed that *Hck* expression was induced by Shh treatment.^39^ In a previous study, *Hck* was identified as a Gli1 binding gene in both CGNPs and medulloblastoma.^38^ To validate that *Hck* is a Shh/Gli target gene, we treated Shh-responsive NIH3T3 cells with Shh-conditioned medium. The *Hck* mRNA (messenger RNA) level was significantly upregulated after Shh treatment. *Gli1* was also upregulated indicating that NIH3T3 cells were activated by Shh (Figure 1a). In CGNP cultures, Shh treatment significantly induced the expression of *Gli1* and *Hck* (Figure 1b). In addition, when we expressed exogenous Gli proteins in NIH3T3 cells, the levels of both *Hck* and another Gli target gene *Ptch1* were significantly increased by Gli1 (Figure 1c). Three Gli transcription factors were evaluated, and *Hck* expression was most sensitive to Gli1 (Figure 1d).

Previous chromatin immunoprecipitation (ChIP)-on-Chip experiments in CGNP and medulloblastoma identified a potential Gli1 binding site ~1.1 kb upstream of the transcription start site of *Hck*; the region harbors a Gli binding motif (Figure 1e, Supplementary Figure S1).^38^ To determine whether Gli1 regulates *Hck* through direct binding, we performed ChIP experiments using antibodies against HA-Gli1 in NIH3T3 cells. ChIP quantitative PCR analyses indicate that Gli1 bound to the upstream enhancer region but not the region surrounding the transcription start site (Figure 1f). To further understand the regulation of the *Hck* gene by Gli1, we cloned 1.7 kb of genomic DNA, including the *Hck* transcription start site and the putative Gli1 binding region, into a luciferase reporter vector. The *Hck* reporter was significantly upregulated upon co-transfection of Gli1 (Figure 1g). Truncation or internal deletion of the Gli1 binding region significantly impaired Gli1-induced reporter activities. Mutation of the 9-bp Gli binding motif resulted in a similar loss of the responsiveness to Gli1 activation (Figure 1g). Taken together, our data indicate that Shh-induced *Hck* expression is mediated by direct binding of Gli1 to the regulatory region upstream of the *Hck* promoter.

### Acute depletion of *Hck* leads to impaired Shh signaling gene expression

**Figure 2:**
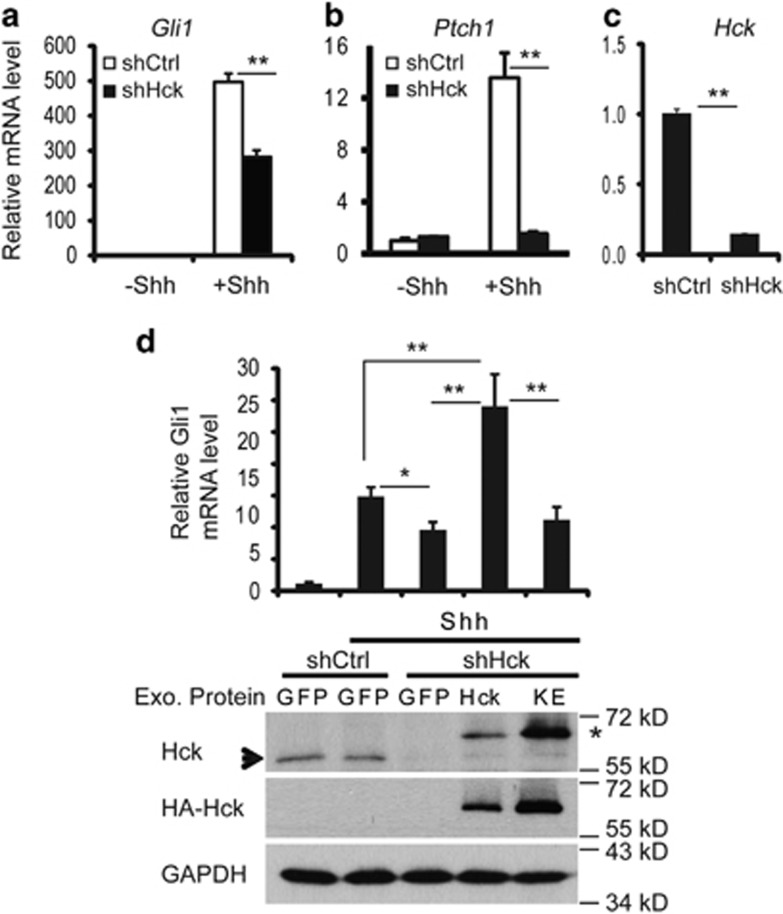
Acute knockdown of Hck leads to impaired Shh signaling-induced gene expression. (**a**–**c**) NIH3T3 cells were infected with lentiviruses expressing scrambled shRNA (shCtrl) or shRNA designed to target *Hck* (shHck). The cells were treated with or without Shh-conditioned medium. mRNA levels of Shh target genes (**a**) *Gli1*, (**b**) *Ptch1* and (**c**) *Hck* were determined by RT–qPCR. (**d**) NIH3T3 cells infected with lentiviruses expressing scrambled shRNA or shHck were co-infected with viruses for expression of Hck, Hck-K269E or GFP control. Upper panel: Endogenous *Gli1* mRNA levels under basal or Shh-stimulated conditions were measured by RT–qPCR. Only wild-type Hck, but not the kinase-inactive Hck mutant (KE), rescued defective Shh-induced *Gli1* expression that resulted from expression of shHck. Lower panel: western blot analyses of endogenous and exogenous Hck in samples analyzed by RT–PCR. An arrow points to the endogenous Hck band and a star indicates HA-Hck. Presented are means plus s.d.; *n*=3. Statistical analyses were performed using the Student's *t*-test; **P<0.01 and *P<0.05. qPCR, quantitative PCR.

To determine whether Hck regulates Shh signaling, we inhibited *Hck* expression using lentivirus-mediated expression of an RNAi agent in NIH3T3 cells. Although *Hck* inhibition did not affect the basal expression of Shh target genes *Gli1* and *Ptch1*, Shh-induced expression of these genes was significantly impaired, indicating that Hck functions in Shh-induced Gli target gene activation (Figures 2a and c). Importantly, the defective gene activation in response to Shh was rescued by an RNAi-resistant human wild-type *Hck* (Figure 2d). Significantly higher Gli1 expression was observed in the presence of exogenous wild-type Hck than in control cells; this is likely owing to overexpression of Hck. Notably, a kinase-inactive form of Hck (Hck-K269E)^40^ did not rescue the defective Gli1 activation that resulted from *Hck* inhibition despite the high levels of expression of the mutant protein (Figure 2d). Thus, Hck is required for Shh signaling in NIH3T3 cells, and its kinase activity is required.

### Hck enhances Gli1-mediated target gene activation to form a positive feedback loop

**Figure 3:**
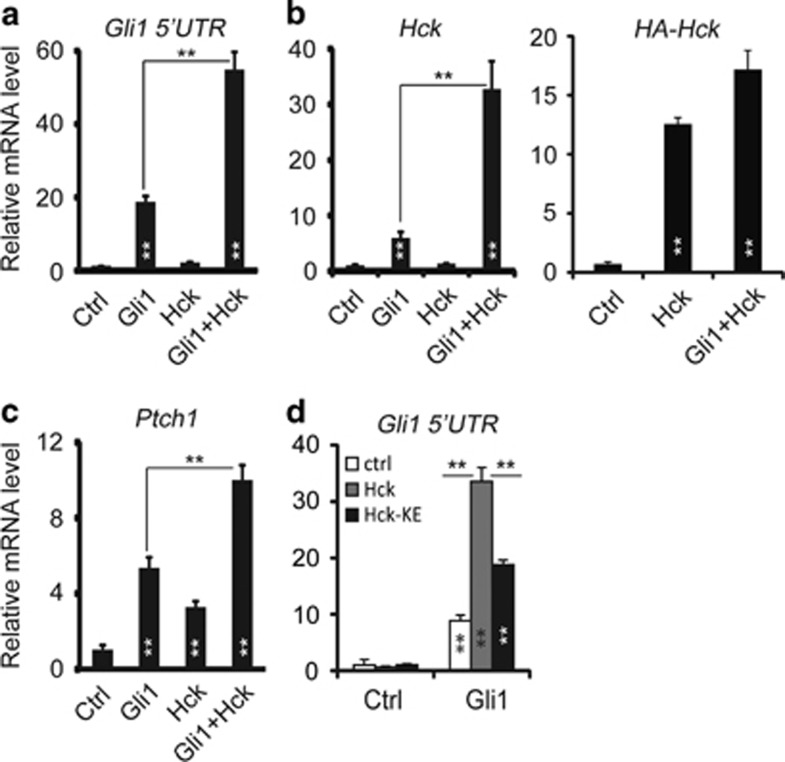
Hck enhances Gli1-mediated target gene activation. (**a**, **b**, **c**) NIH3T3 cells were infected with lentiviruses expressing Gli1 alone, Hck alone or the two together. Levels of Shh target genes (**a**) *Gli1*, (**b**) *Hck* and (**c**) *Ptch1* were determined by RT–qPCR. Endogenous *Gli1* levels were measured using primers in the 5′-UTR to distinguish this message from that of the exogenous Gli1. Exogenous human *Hck* levels were measured using primers specific to human genes. (**d**) Hck kinase activity is required for maximum activation of Gli1. As in **a**, Gli1-induced expression of endogenous *Gli1* was measured in the presence of wild-type Hck or Hck-K269E kinase-dead mutant (Hck-KE). Presented are means plus s.d.; *n*=3. Statistical analyses were performed using the Student's *t*-test; **P<0.01. qPCR, quantitative PCR.

To delineate how Hck regulates Gli-mediated gene transcription in response to Shh signaling, we co-expressed Gli1 and Hck in NIH3T3 cells. Exogenous Gli1 activated the expression of endogenous Shh target genes *Ptch1*, *Gli1* and *Hck* as expected, whereas Hck alone had a moderate or no effect on the target gene expression. Expression levels of exogenous human *Hck* and endogenous *Hck* were distinguished by using specific PCR primers in human and mouse *Hck* genes, respectively. Interestingly, co-expression of Hck and Gli1 significantly enhanced Gli1 activator activities. All Shh target genes tested were expressed at higher levels in the presence of exogenous Hck than in the presence of Gli1 alone (Figures 3a and c). Interestingly, the kinase-dead Hck-K269E mutant protein also enhanced Gli1-mediated target gene induction albeit with less fold change than wild-type Hck (Figure 3d), suggesting that besides the kinase activities, kinase-independent mechanisms also exist to activate Gli1. Thus, as *Hck* is a target gene of Gli1 and it encodes an activator of Gli1, Hck and Gli1 form a positive feedback loop that amplifies Shh signaling outcomes.

### Hck phosphorylates multiple residues in Gli1 through direct binding

**Figure 4:**
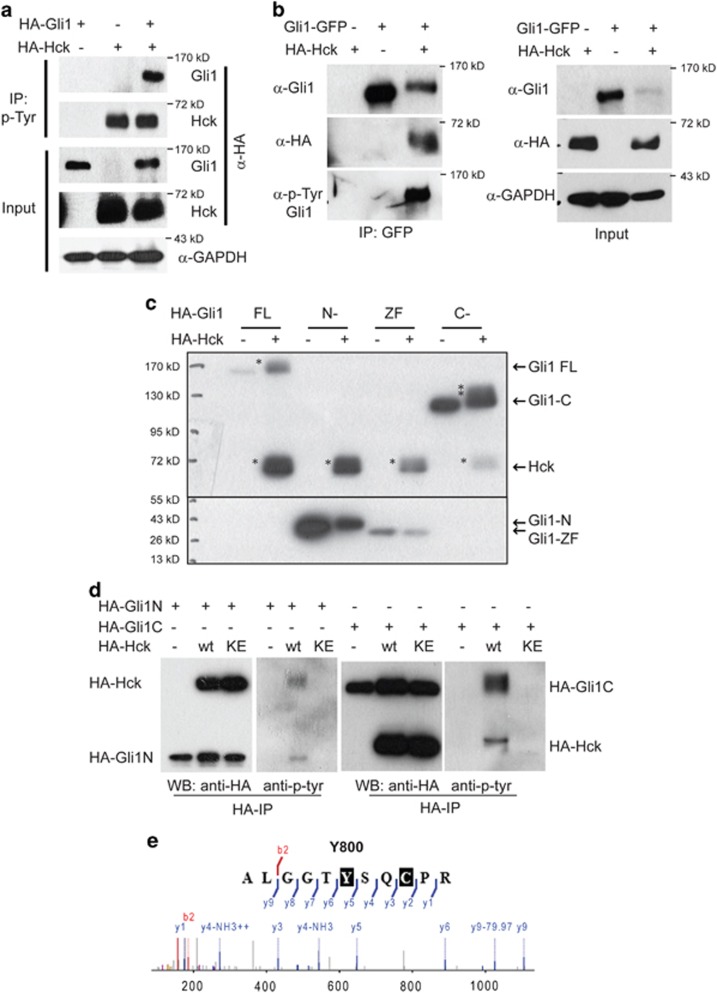
Hck phosphorylates multiple residues in Gli1 through direct binding. (**a**) Gli1 is Tyr phosphorylated in the presence of exogenous Hck. NIH3T3 cells were transiently transfected with constructs expressing HA-tagged Gli1 alone, Hck alone or the two together. Cell lysates were immunoprecipitated using 4G10 antibody, followed by western blot using antibody against HA tag. (**b**) Gli1 co-precipitates with Hck. NIH3T3 cells were transfected with constructs for expression of GFP-Gli1, HA-Hck or the two together. Antibodies against GFP were used for the immunoprecipitation and antibodies against Gli1, HA and phospho-Tyr (4G10) were used for western blot. HA-Hck was co-precipitated with GFP-Gli1. Gli1 was Tyr phosphorylated in the presence of Hck as shown by the 4G10-positive band. GAPDH was used as a loading control. (**c**) Western blot analyses of Hck phosphorylated human Gli1 fragments (FL, full-length; N-, N-terminal fragment (1-231 aa); ZF, zinc-finger domain (232-410 aa); C-, C-terminal fragment (411-1106 aa)) separated on an SDS–PAGE gel. NIH3T3 cells were co-transfected with constructs for expression of Gli1 fragments and HA-Hck. Asterisks indicate the phosphorylated bands; arrows point to the corresponding non-phosphorylated protein bands. (**d**) Both the N- and C-terminal fragments of Gli1 were Tyr phosphorylated in the presence of Hck. NIH3T3 cells were transfected with constructs for expression of HA-tagged Gli1 fragments and HA-Hck or the kinase-inactive Hck-K269E. Antibodies against HA were used for the immunoprecipitation and antibodies against HA, and phospho-Tyr (4G10) were used for western blot. (**e**) HPLC-MS/MS spectrum of phosphopeptide ALGGTY(p)SQCPR that contains Y800. The ion peak labeled with minus 79.97 (H_3_PO_4_ mass) serves to confirm the Y800 phosphorylation.

Hck is a Src family tyrosine kinase; Hck phosphorylates itself and other substrate proteins.^41^ To examine whether Hck can phosphorylate Gli1, we expressed HA-tagged Gli1 and Hck individually or simultaneously in NIH3T3 cells, then immunoprecipitated proteins phosphorylated at Tyr using the antibody 4G10 specific for phosphorylated Tyr residues. Consistent with the previously reported auto-phosphorylation function of Hck,^41^ HA-Hck migrated at ~59 kD was detected in the proteins precipitated with 4G10 from cells expressing exogenous Hck. Without exogenously expressed Hck, Gli1 was not precipitated with 4G10. With co-expressed Hck, HA-Gli1 migrated at ~150 kD was detected in the 4G10 precipitations (Figure 4a), suggesting that Gli1 is phosphorylated by Hck. To determine whether Hck interacts with Gli1, we performed co-immunoprecipitation experiments. NIH3T3 cells were transfected with vectors for expression of Gli1-GFP and/or HA-Hck. Cell lysates were immunoprecipitated with antibodies against the GFP tag. HA-Hck was co-immunoprecipitated with Gli1-GFP. Gli1-GFP was Tyr phosphorylated in the presence of HA-Hck as it reacted with the 4G10 antibody (Figure 4b).

In the Gli1 protein, there are 32 Tyr residues that are conserved between mouse and human (Supplementary Figure S2). To determine which residues can be phosphorylated by Hck, we evaluated phosphorylation of the full-length Gli1 protein, the N-terminal region, the zinc-finger domain and the C-terminal fragment expressed in NIH3T3 cells. Using a western blot analysis, we observed that the full-length Gli1 and the C-terminal fragment were phosphorylated as indicated by multiple slow-migrating bands (Figure 4c, indicated by asterisks). The tyrosine phosphorylation in both the N- and C-terminal regions of Gli1 was also detected with 4G10 antibody western blot following immunoprecipitation with anti-HA antibodies (Figure 4d). These results indicate that multiple Tyr residues in the N- and C-terminal domains of Gli1 are likely phosphorylated by Hck. This is consistent with previous findings that Gli proteins are relatively unstructured and interact with other proteins through both N- and C-terminal domains.^27, 42^ Therefore, Hck likely regulates Shh signaling output by interacting and phosphorylating Gli1.

To identify the potential Hck-targeted Tyr phosphorylation sites in Gli1, HA-Gli1 co-expressed with Hck in NIH3T3 cells were immunoprecipitated with anti-HA antibodies and separated on SDS–PAGE (SDS polyacrylamide gel electrophoresis) gels. Gli1 proteins in gel slices were digested, extracted and subjected for LC/MS/MS. Within 23 Gli1 peptides recovered (26.3% coverage), we identified Y800 as a phosphorylated residue with high confidence (Figure 4e). Y800 is a potential Hck target site in Gli1. However, deleting Y800 did not diminish the Gli1 Tyr phosphorylation (Supplementary Figure S3). This observation confirmed that Gli1 is Tyr phosphorylated at multiple sites including Y800.

### Hck disrupts Gli–Sufu interactions

**Figure 5:**
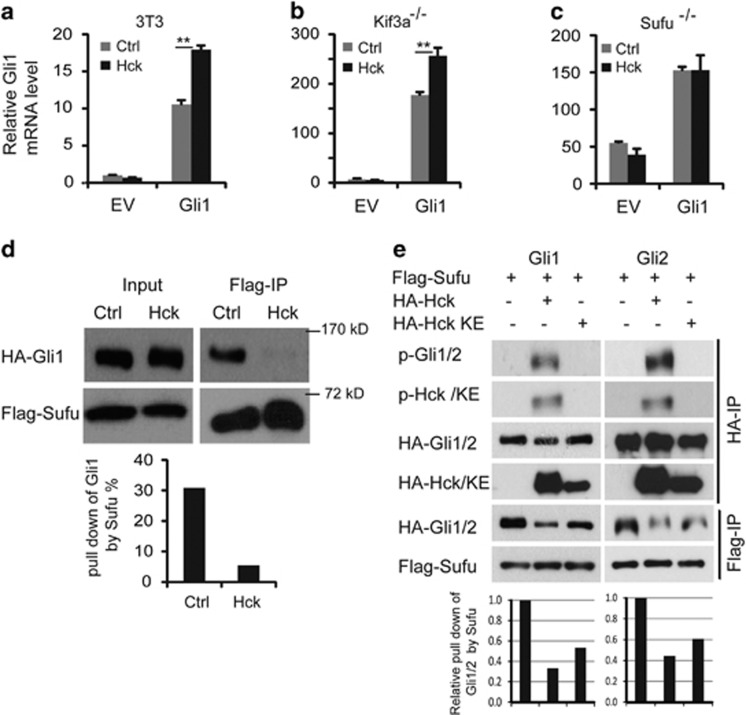
Hck disrupts Gli–Sufu interactions. (**a**–**c**) Hck activates Gli1 downstream of Kif3a in a Sufu-dependent fashion. (**a**) NIH3T3 cells, (**b**) immortalized *Kif3a*^*−/−*^ MEFs and (**c**) *Sufu*^−^^*/−*^ MEFs were co-infected with a control or with lentiviruses designed to express Gli1 and/or Hck. Endogenous *Gli1* levels measured using primers in the 5′-UTR were determined by RT–qPCR. Presented are means plus s.d.; *n*=3. Significance was determined by Student's *t*-test; **P<0.01. (**d**) Hck disrupts Gli1–Sufu interactions. NIH3T3 cells were transfected with constructs expressing HA-Gli1 and Flag-tagged Sufu in the presence or absence of exogenous HA-Hck. Cell lysates were precipitated with anti-Flag antibodies and western blotted with antibodies against HA or Flag. The western blot bands were quantified with NIH Image J software and the percentage of Gli1 precipitated with Flag-Sufu was compared with the input. (**e**) NIH3T3 cells were transfected with constructs expressing HA-Gli1/2 and Flag-Sufu in the presence of exogenous HA-Hck or kinase-inactive Hck-K269E. Cell lysates were precipitated with anti-Flag or anti-HA antibodies and western blotted with antibodies against HA, Flag or 4G10 antibodies. Quantifications of western blot are shown below. qPCR, quantitative PCR.

Gli1 activities can be regulated at several different steps. Two critical steps are the activation in cilia and the inhibition by Sufu. Kif3a is required for the formation and function of primary cilia in transducing the Shh signal.^25, 43^ Interestingly, in *Kif3a*^−/−^ MEF cells, *Gli1* transcriptional activation was still enhanced by exogenous Hck (Figures 5a and b), indicating that Hck functions downstream of Kif3a. On the contrary, in *Sufu*^−/−^ MEF cells,^44^ Hck failed to enhance Gli1 activities in inducing endogenous *Gli1* expression (Figure 5c). This result indicates that Hck functions at the same level or downstream of Sufu.

Sufu interacts tightly with Gli activators and inhibits their activities at several levels.^22, 24, 25, 26, 42^ We next examined whether Hck affects the interaction between Gli1 and Sufu. In NIH3T3 cells expressing tagged Gli1 and Sufu, in the absence of exogenous Hck, ~30% of Gli1 co-immunoprecipitated with Sufu. However, in the presence of Hck, only ~5% of the Gli1 co-precipitated with Sufu (Figure 5d). In a gel with better resolution, it appeared that only unphosphorylated Gli1 co-precipitated with Sufu (Supplementary Figure S4). These results suggest that the interaction between Gli1 and Hck or the phosphorylation of Gli1 by Hck disrupts Sufu–Gli1 interaction. To determine whether the kinase activity of Hck is required, we performed the experiments with the kinase-inactive Hck-K269E. Interestingly, co-expressing Hck-KE also significantly reduced the co-immunoprecipitation efficiency of Gli1 with Sufu (Figure 5e). Thus, kinase activity-independent mechanisms could be largely responsible for Hck function in disrupting Gli1–Sufu interaction, possibly through Hck–Gli1 interactions. Similar to Gli1, Gli2 is also inhibited by Sufu. In NIH3T3 cells, both Hck and Hck-KE mutant proteins significantly reduced Gli2 co-immunoprecipitation with Sufu (Figure 5e). Thus, Hck could also disrupt Gli2–Sufu interactions. These results indicate that one mechanism that Hck uses to activate Gli1 and possibly Gli2 is to release Gli proteins from Sufu inhibition.

### Hck is highly expressed in Shh-type medulloblastoma and required for tumor cell growth

**Figure 6:**
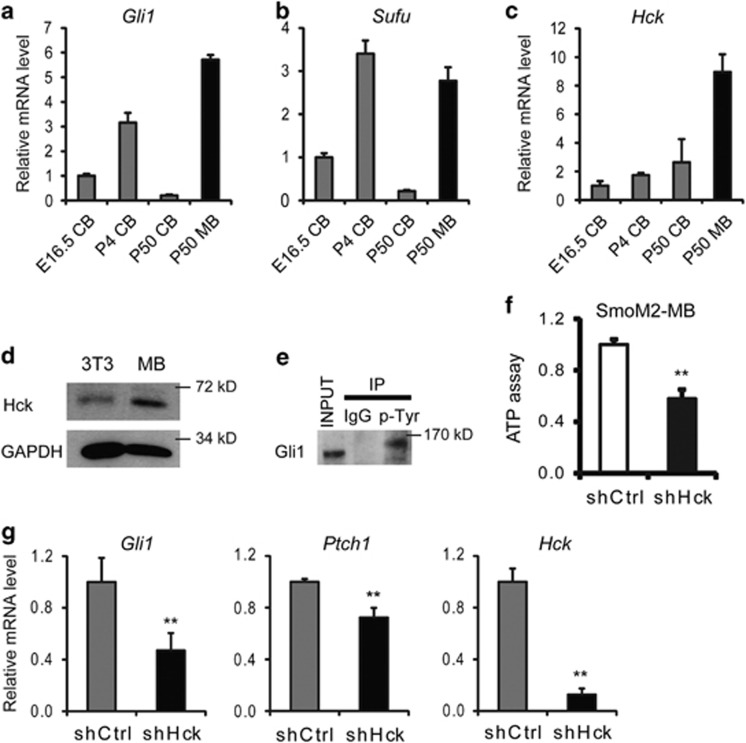
Hck is highly expressed in SmoM2-induced medulloblastoma cells and required for its growth. (**a**–**c**) Expression levels of (**a**) *Gli1*, (**b**) *Sufu* and (**c**) *Hck* in cerebellum (CB) during different developmental stages and in SmoM2-induced medulloblastoma (MB) are shown. (**d**) The levels of Hck protein in NIH3T3 cells and in SmoM2 medulloblastoma were determined by western blot. (**e**) Endogenous Gli1 protein in medulloblastoma is Tyr phosphorylated. SmoM2 medulloblastoma lysates were precipitated with 4G10 antibody against phospho-Tyr and probed by western blot using antibodies against Gli1. (**f**) Cultured SmoM2 medulloblastoma cells were infected with lentiviruses expressing control (scrambled shRNA) or shHck. The survival rates of Hck-deficient tumor cells relative to the control cultures were measured using an ATP cell viability assay. (**g**) RT–PCR analyses of the expression levels of *Gli1*, *Ptch1* and *Hck* in cultured SmoM2 medulloblastoma cells with *Hck* RNAi knockdown. Presented are means plus s.d.; *n*=3. Significance was determined by Student's *t*-test; **P<0.01.

During early postnatal cerebellum development (postnatal day 0 (P0) to P14 for mouse), Shh signaling induces rapid proliferation of CGNPs, which then differentiate into granule neurons. Mutations such as *Ptch1* loss-of-function or *Smo* gain-of-function lead to constitutively active Shh signaling, which results in CGNP over-proliferation and medulloblastoma.^13^ A mouse model of medulloblastoma with an activating SmoM2 mutant transgene closely resembles human Shh-type medulloblastomas.^45^ The high *Gli1* levels that we observed in P4 cerebellum and in medulloblastoma samples indicate active Shh signaling during cerebellum development and in Shh-type medulloblastoma formation (Figure 6a). High levels of *Sufu* were also found in developing cerebellum and in medulloblastoma (Figure 6b), indicating inhibition of Shh/Gli1 activities despite activation of Shh signaling. Interestingly, both mRNA and protein levels of Hck were significantly higher in medulloblastoma compared with normal cerebellum and NIH3T3 cells (Figures 6c and d, Supplementary Figure S5), which may contribute to the abnormally high Shh/Gli1 activities in Shh-type medulloblastoma and the uncontrolled proliferation of these cells. Indeed, we found that endogenous Gli1 is Tyr phosphorylated in medulloblastoma as indicated by 4G10 antibody immunoprecipitation (Figure 6e). Importantly, inhibition of *Hck* expression mediated by RNAi in cultured SmoM2 medulloblastoma cells significantly decreased the survival and growth of cancer cells as indicated by cell viability assays (Figure 6f). Key Shh target genes such as *Gli1* and *Ptch1* were significantly decreased upon Hck inhibition (Figure 6g). Our data indicate that high level of Hck is required for maximum Gli1 activities and oncogenic functions in medulloblastoma. Disrupting Gli1–Hck feedback loop would be a promising treatment strategy for Shh-type medulloblastoma.

## Discussion

**Figure 7:**
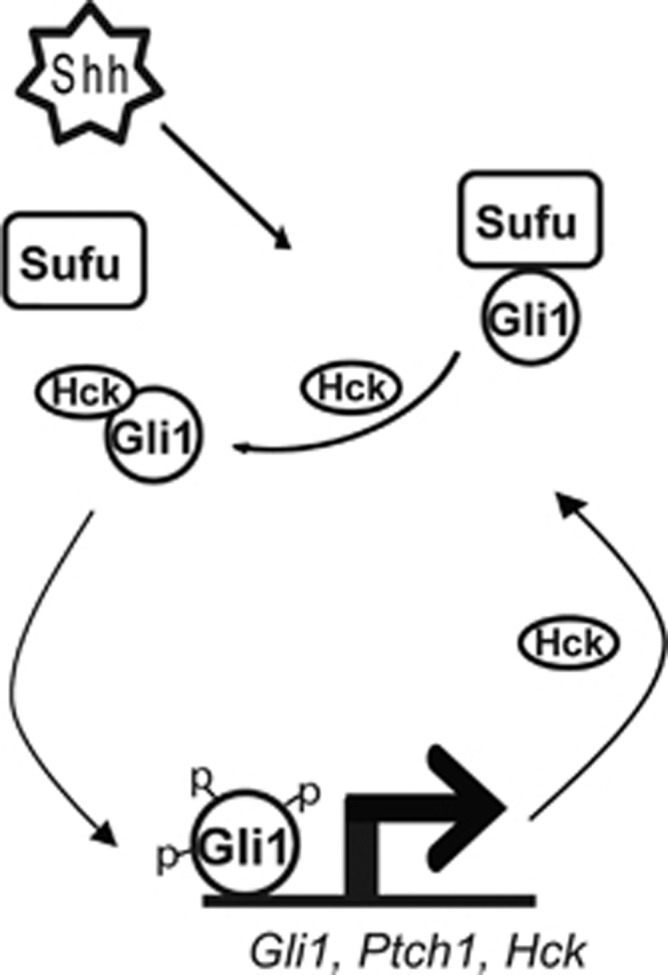
A model representing the positive feedback loop formed by Hck and Gli1 in activating Shh target genes. Active Gli1 in response to Shh signaling induces the expression of Shh target genes including *Hck*, which encodes a tyrosine kinase that could enhance Gli1 transcription activator functions. Hck disrupts the interaction between Gli1 and its inhibitor Sufu, possibly through Hck–Gli1 interactions. Hck could phosphorylate Gli1 and the Tyr phosphorylation of Gli1 further enhances the Gli1 activities. Thus the positive feedback loop formed by Hck and Gli1 amplifies Shh signaling outputs.

In the past two decades, significant progress has been made in understanding how Shh signaling contributes to normal development and cancer progression. Recent genomic studies on the basis of the transcription profiles have shown that ~25% of medulloblastoma cases are characterized by active Shh signaling. In this study, we demonstrate that a tyrosine kinase Hck forms a positive feedback loop with the transcription activator Gli1 to amplify Shh signaling outputs; this feedback loop contributes to the tumorigenic function of Shh signaling (Figure 7). We found that *Hck* is a direct Shh target gene that is sensitive to Gli1 activation. We provide evidence that Hck enhances Gli1 activities and that this function of Hck is mediated by both phosphorylation of Gli1 and kinase-independent activities. High level of Hck disrupts the interaction between Gli1 and its inhibitor Sufu with kinase activity-independent mechanisms. Importantly, Hck is highly expressed in Shh-type medulloblastoma and required for tumor cell growth. Thus, disrupting the Gli1–Hck feedback loop may inhibit progression of Shh-type medulloblastoma.

In this study, we demonstrate that Gli1 is activated by novel mechanisms through tyrosine phosphorylation and interaction with a tyrosine kinase. We showed that tyrosine kinase Hck activates Gli1 and the kinase activity is required for its maximum effect. In medulloblastoma in which Gli1 is highly expressed and activated, we observed Tyr phosphorylation of endogenous Gli1. There are 32 conserved Tyr residues throughout the Gli protein. Our truncation analyses indicated that multiple residues are likely phosphorylated by Hck. We identified Y800 in human Gli1 as a potential Hck target site. This is consistent with a report from the PhosphoSitePlus database that the conserved Y798 in rat was phosphorylated in ischemic esophagus. In Gli1, Y800 is located in the proline-rich region that may be important in both the active Gli1 conformation and its interactions with other regulators. However, it is likely that additional functional Tyr residues are phosphorylated by Hck. Notably, Y121 in the SYGH Sufu binding motif is important for maintaining the hydrophobic interacting surface between Gli1 and Sufu.^46, 47^ Although we confirmed the importance of Y121 in Gli1–Sufu interaction by mutagenesis analyses (data not shown), we did not observe the phosphorylation of the Tyr by mass spectrometry. Thus, analyses of combinations of Tyr mutations or more targeted mass spectrometry studies may reveal the entire Gli1 phosphorylation pattern that activates Gli1 function. In addition, our data do not definitively exclude the possibility that Hck phosphorylates other Gli1 regulators and activates Gli1 indirectly.

In the study, we observed that Hck could activate Gli1 through kinase-independent activities as both wild-type and kinase-inactive Hck could activate Gli1-mediated target gene activation and disrupt the interaction between Gli1 and its inhibitor Sufu. As Hck interacts with Gli1, it is possible that high levels of Hck could compete with Sufu for interaction. Similarly, Hck could also interrupt Gli2–Sufu interactions, which may contribute to Hck functions in amplifying Shh signaling outputs. Although Sufu is a major Gli1 inhibitor, Hck may further activate Gli1 through phosphorylation as the kinase activity is required for maximum Gli1 activation by Hck. Hck may phosphorylate Gli1 to promote its transcription activities or to affect its interaction with other regulators or co-factors.

Although both Gli1 and Hck are important for Shh target gene activation, they are not required for normal development.^19, 37, 48, 49^ During normal development, Gli2 is sufficient for Shh-induced gene activation.^19^ Gli1 is required for medulloblastoma formation and is more potent than Gli2 at inducing cell transformation.^50^ Thus Gli1 activity is important for the abnormally high transcription output of Shh signaling that are required for tumorigenesis. We speculate that Hck is induced by high levels of Gli1 and that the positive feedback loop with Gli1 operates only in the presence of highly active Shh signaling such as that observed in medulloblastoma. Our data support this hypothesis: (1) Hck transcriptional activation was much more sensitive to exogenous Gli1 than to Gli2 or Shh stimulation (Figure 1, 500-fold by Gli1 versus 50-fold by Gli2 and 5-fold by Shh). (2) Hck was highly expressed in Shh-type medulloblastoma where Gli1 was highly expressed and tyrosine phosphorylated. Hck levels in normal cerebellum and Shh-independent medulloblastoma were relatively low (Figures 6c, Supplementary Figure S5). (3) High levels of Hck interrupt Gli1–Sufu interactions and activate Gli1-mediated target gene activation. (4) Inhibition of *Hck* expression significantly inhibited Shh target gene expression and medulloblastoma growth (Figures 6f and g). Taken together, our results indicate that increased Hck expression in medulloblastoma induced by abnormally active Shh signaling enhances Gli1 oncogenic activities and contributes to tumor growth. As some of our experiments only examined the *Hck* RNA levels, an investigation of the Hck protein levels in medulloblastoma may further strengthen our conclusions.

As Hck is a novel enhancer for Gli1 oncogenic activities in medulloblastoma, Hck is a potential treatment target. A kinome study in medulloblastoma indicated high levels of Src family kinase (SFK) activities.^51^ SFK inhibitors effectively inhibit medulloblastoma cell growth;^52^ however, it is not clear whether these medulloblastoma cells are of the Shh subtype and little is known about how other SFKs regulate Shh signaling. Src has been shown to inhibit primary cilia growth^53^ and is expressed at low levels in Shh-type medulloblastoma (Supplementary Figure S5A), and thus may be an inhibitor of Shh signaling and Shh-type medulloblastoma growth. Thus SFKs may have opposing functions in Shh-induced tumors. As Hck may also enhance Gli1 activities with kinase-independent activities, specific inhibitors disrupting the Hck–Gli1 feedback loop would be more effective in inhibiting Shh-type medulloblastoma cancer progression than general kinase inhibitors.

In summary, our study identified the tyrosine kinase Hck as both a target of Gli1 and a regulator of Gli1 activation. The positive feedback loop formed by Gli1 and Hck amplifies Shh signaling output and contributes to medulloblastoma cell growth. Inhibiting Hck activities or disrupting the Hck–Gli1 feedback loop may be effective approaches for the treatment of Shh-type medulloblastoma and possibly other cancers with elevated Shh/Gli1 activities.

## Materials and methods

### Mice

The *SmoM2*^45^ and *CAG-CreER*^54^ transgenic mice were purchased from Jackson Laboratory. *SmoM2 CAG-CreER* mice develop medulloblastoma spontaneously at a frequency of 40%.^45^ The mice were maintained on a mixed genetic background at UT Southwestern Medical Center Animal Facility.

### Cell line, primary CGNP and medulloblastoma cell cultures

NIH3T3 cells (an immortalized MEF cell line), immortalized *Kif3a*^−^^*/−*^ MEF cells and *Sufu*^*−/−*^ MEF cells were maintained in DMEM (Dulbecco's Modified Eagle's medium) containing 10% fetal bovine serum. The *Kif3a*^*−/−*^ cells were provided by Dr PT Chuang.^25^ *Sufu*^*−/−*^ MEFs were kindly provided by Dr R Toftgard.^44^ Primary CGNP cultures were derived from dissociated P4 mouse cerebella and cultured in DMEM/F12 media containing 25 mm KCl, N_2_ and 10% fetal bovine serum, as previously described.^27^ For Shh stimulation, Shh-conditioned medium produced from Shh-CM 293 T cells^55^ was added at a 1:20 dilution to MEF and CGNP cultures. NIH3T3 cells were treated with Shh in low-serum media 24 h before harvesting. Primary tumor cells were derived from dissociated SmoM2 medulloblastoma and cultured in the media containing DMEM/F12, B27, N_2_, EGF and FGF2. The ATP assay for cell viability analysis was carried out as described.^39^

### Luciferase reporter assay

Transient transfection and luciferase assays were done in NIH3T3 cells essentially as described.^56^ Co-transfection of the vector phRL-TK Renilla (Promega, Fitchburg, WI, USA) allowed normalization of transfection efficiencies. The normalized data were expressed as multiples of the activity of the 1.7 kb Hck promoter reporter in pGL3basic.

### Plasmid construction, virus preparation and transfection/infection

The shRNA sequence targeting mouse *Hck* (5′-TACCATTGTGGTCGCACTGTA-3′) was cloned into the PLKO lentiviral vector. The PLKO construct with a scrambled shRNA sequence was used as a negative control. Lentiviral vector pSin4-EF2-IRES-Puro was used to generate expression constructs for 3 × HA- or GFP-tagged human Gli1/2/3 and Hck. Lentiviruses were prepared according to a previously described procedure.^27^ PolyJet (Signagen, Gaithersburg, MD, USA) was used for plasmid transfection of cultured cells. Attached cultured cells were infected at a multiplicity of infection of 5 for 24 h in media with 8 μg/ml polybrene.

### Immunoblotting

For immunoblotting, cells or tissues were lysed in RIPA buffer (50 mm Tris, pH 8, 150 mm NaCl, 0.05% SDS, 0.5% DOC, 1% NP-40), and cell lysates were separated on SDS–PAGE gels. Antibodies used for western blot recognized Hck (#06-833, Millipore, Billerica, MA, USA), HA (HA-7, Sigma-Aldrich, St Louis, MO, USA), anti-phospho-tyrosine (4G10, Millipore), Gli1 (#2643, Cell Signaling, Danvers, MA, USA), GFP (A11122, Life Technologies, Carlsbad, CA, USA) and GAPDH (G9545, Sigma-Aldrich). HRP-conjugated secondary antibodies were purchased from Jackson ImmunoResearch (West Grove, PA, USA). GAPDH was detected as a loading control.

### Mass spectrophotometry analyses of Gli1 Tyr phosphorylation

HA-Gli1 co-expressed with Hck in NIH3T3 cells were immunoprecipitated with anti-HA antibody and separated on SDS–PAGE gels. Coomassie Blue-stained Gli1 band was isolated and subjected for mass spectrophotometry analyses. Proteins from the gel slice were digested, extracted and analyzed by LC/MS/MS (UT Southwestern Medical Center Proteomic Core Facility, Dallas, TX, USA). Peptide identification was performed using the X!Tandem^57^ and open MS search algorithm (OMSSA)^58^ search engines against the mouse protein database from Uniprot, 23 Gli1 peptides were identified, which covers 26.3% of the Gli1 protein. Phosphorylation sites were localized using ModLS PTM Localization and confirmed by manual interpretation.

### Co-immunoprecipitation experiments

Experiments were performed essentially as described previously.^27^ Antibodies were against the HA tag (ab9110, Abcam, Cambridge, UK), Flag-tag (F1802, Sigma), anti-phospho-tyrosine (4G10, Millipore) and GFP (A11122, Life Technologies). Shh-responsive NIH3T3 cells were transiently transfected with plasmids expressing HA- or GFP-tagged proteins using PolyJet (Signagen). Mock transfection was used as the negative control. Cells were collected 24–48 h after transfection and were lysed with co-IP Lysis Buffer (50 mm Tris, pH 8.0, 150 mm NaCl, 1 mm EDTA, 1% Triton X-100, with protease inhibitor freshly added). Cell lysates were snap-frozen in liquid nitrogen and then thawed on ice followed by sonication to facilitate cell lysis. After centrifugation, appropriate antibodies were added to pre-cleared cell lysate and incubated at 4 °C overnight. Samples were incubated with protein A beads (GE Healthcare, Dallas, TX, USA) for 1 h; beads were washed with co-IP buffer four times. Precipitated proteins were eluted by boiling in 2 × Sample Buffer before SDS–PAGE and western blot analysis.

### ChIP assay

ChIP experiments were performed as described previously.^27^ Dissociated cells were crosslinked with PFA, and DNA was sonicated to fragments (200–1000 bp). Antibody against HA (ab9110, Abcam) was used in the precipitation step. Rabbit IgG was used as a negative control. Precipitated DNA was purified and subjected to real-time PCR.

### Real-time quantitative PCR

RNA from cells or tissues was extracted with TRIZOL (Invitrogen, Carlsbad, CA, USA). Complementary DNAs were synthesized by reverse transcription using Iscript (Bio-Rad, Hercules, CA, USA), followed by PCR or quantitative PCR analysis. A Bio-Rad real-time PCR system (C1000 Thermal Cycler) was used for quantitative PCR. Levels of *GAPDH* mRNA were used to normalize input RNA. Graphics shown are representative of experiments performed in triplicate. Sequences of PCR-primers used are listed in Supplementary Table S1.

### Statistical analysis

Data are expressed as means plus s.d. The error bars are standard deviations of three analyses of one representative experiment. Each experiment was repeated at least three times. Statistical analyses were performed using a two-tailed, unpaired Student's *t*-test.
