# Supplementary material for: A positive feedback loop between Gli1 and tyrosine kinase Hck amplifies shh signaling activities in medulloblastoma
Source: Oncogenesis. 2015 Nov 30;4(11):e176–. doi: 10.1038/oncsis.2015.38 (PMC4670963; doi:10.1038/oncsis.2015.38)

Supplementary Information

Supplementary Figure Legends

Figure S1. Nucleotide sequence of the 1.7-kb genomic DNA around the *Hck* transcription start site. Positions are given relative to the transcription start site (TSS, +1) of the *Hck* gene. The boxed regions indicate ChIP-qPCR products. The potential Gli binding motif is indicated.

Figure S2. Comparison of human and mouse Gli1 protein sequences. The conserved Tyr residues are marked with upper case “Y” below the sequence. The lower case “y” indicates the tyrosine residues present only in mouse or human Gli1. The zinc-finger domain is indicated.

Figure S3. Phosphorylation of Gli1 mutants by Hck. (A) Map of Gli1 constructs relative to the full-length Gli1 protein. Several conserved Tyr residues are marked. (B) NIH3T3 cells were co-transfected with constructs expressing Hck and different HA-tagged Gli1 proteins. Cell lysates were precipitated with 4G10 antibody and probed with anti-HA. Phosphorylation of all Gli1 constructs was detected.

Figure S4. Sufu interacts with non-phosphorylated Gli1 proteins. NIH3T3 cells were transfected with constructs expressing HA-Gli1 and Flag-Sufu proteins in the presence or absence of exogenous HA-Hck. Cell lysates were precipitated with anti-Flag antibodies and antibodies against HA or Flag were used for western blot. Phosphorylated Gli1 bands (which migrate more slowly) were detected in the input protein lysate (marked by a *) but not in the Flag-precipitated compartment in the presence of exogenous Hck.

Figure S5. Hck is highly expressed in Shh-type medulloblastoma (A) Microarray analyses (NCBI Geo Datasets GSE9299) indicate that both *Gli1* and *Hck* but not *Src* mRNA levels are higher in SmoM2 induced medulloblastoma than in normal cerebellum controls. (B) Microarray analyses (GSE34126) indicate that *Hck* mRNA levels in mouse *Ptch1*-null Shh-type medulloblastomas are higher than those in normal stem cells and those in Myc-driven Group 3 like medulloblastomas.

Supplementary Tables

Table S1 Sequences of PCR-Primers

Gli1F	GGTCTCGGGGTCTCAAACTGC
Gli1R	CGGCTGACTGTGTAAGCAGAG
Gli15UTRF	GCTGGAGGTCTGCGTGGTAGAG
Gli15UTRR	GAGGAGGAGGAGGAAAGAGAGATCC
HckhmF	TCACTGGTGTGTAAGATTGCTG
HckhmR	GGGTTTGACATACCTGGGTAAG
HckmF	CAAGTCTTCGTCGCTTGCTC
HckmR	CGGAGGAACCTGGACTTCAC
Ptch1F	GAAGCCACAGAAAACCCTGTC
Ptch1R	GCCGCAAGCCTTCTCTAGG
SufuF	ATGGCCGGCACTTCACCTACAAG
SufuR	GGCCAGCTGTACTCTTTGGGAAG
GapdhF	GTGGTGAAGCAGGCATCTGA
GapdhR	GCCATGTAGGCCATGAGGTC

Figure S1

```
-1575   GCTGCCTTTA TTGGCTTCTT TCCCATCTCA ATCTCCTTCA CTCAGTAGAT AACAAGCATT GTTTACTGCT ACACAGACTA
        CGACGGAAAT AACCGAAGAA AGGGTAGAGT TAGAGGAAGT GAGTCATCTA TTGTTCGTAA CAAATGACGA TGTGTCTGAT
-1495   ATTTCACTTC TGTGTCCAAG TCAATTAAAC CTGTTCAAGA CTCGGTCTAT TTTTAGAAAT AGGGCATGAA TATATTAAAA
        TAAAGTGAAG ACACAGGTTC AGTTAATTTG GACAAGTTCT GAGCCAGATA AAAATCTTTA TCCCGTACTT ATATAATTTT
-1415   TACATAGCAA ACACATTAGT GTTGGGTGAC TGGGATTTCT ACGTGGGCTG GGGACCAGGC TGTGTAGGAG GCAGCTCTGG
        ATGTATCGTT TGTGTAATCA CAACCCACTG ACCCTAAAGA TGCACCCGAC CCCTGGTCCG ACACATCCTC CGTCGAGACC

                                                                                 EcoRI
-1335   TTGTCAGAAC TGCTACTGGG CCTAGCGGGT GGAACTCTAG AGCTGCTAAC ACCGCTCGCG CTCAGAATTC ATACTTAAAA
        AACAGTCTTG ACGATGACCC GGATCGCCCA CCTTGAGATC TCGACGATTG TGGCGAGCGC GAGTCTTAAG TATGAATTTT
-1255   CATAGTACTG CTAAACGCAT GCCTACATGC ACGCGTAAGC GAATGAATGA GAGATACTGG TGGTCTGGCC TGGCTAGAGG
        GTATCATGAC GATTTGCGTA CGGATGTACG TGCGCATTCG CTTACTTACT CTCTATGACC ACCAGACCGG ACCGATCTCC

                                 PstI
-1175   AGGGAGGACT CCTGTACGTT TCTGCAGGTG TGTGTTGCAG TTTAGAGAGT GTTGGTCCCT GTCGTTTTGA ATGATGTCTC
        TCCCTCCTGA GGACATGCAA AGACGTCCAC ACACAACGTC AAATCTCTCA CAACCAGGGA CAGCAAAACT TACTACAGAG
                                                                                 Gli binding site
-1095   AGGCTGAGTG GAATATGCCA TGCAAACAGG ACCTCAGTGG TCTGCCAGTA AGACTTCCTC TCTTCAACTT GGGAGGTCCT
        TCCGACTCAC CTTATACGGT ACGTTTGTCC TGGAGTCACC AGACGGTCAT TCTGAAGGAG AGAAGTTGAA CCCTCCAGGA
-1015   CTGAGCAGTC CCATATTCAG CCCAGAATAA GGCAAAGAAC TCCCGCGGGC GCCAGACTTT GCATGGAGTT TGAATAAGCG
        GACTCGTCAG GGTATAAGTC GGGTCTTATT CCGTTTCTTG AGGGCGCCCG CGGTCTGAAA CGTACCTCAA ACTTATTCGC
 -935   GTGGGTGGGC GGGTGGAGGG AAAACCGCTG CATGTGGGAG GGACCGAAGT GGAAGGGCGG TGGAGACCCA CCAGAATCCT
        CACCCACCCG CCCACCTCCC TTTTGGCGAC GTACACCCTC CCTGGCTTCA CCTTCCCGCC ACCTCTGGGT GGTCTTAGGA
 -855   AGAGGGAGAG GTTAGACTTA TCAGGGCGTG GTTAGACTTA AAGGGAGCTA CATTCCTTCT GCGGCTGGTT TAGGATCTTT
        TCTCCCTCTC CAATCTGAAT AGTCCCGCAC CAATCTGAAT TTCCCTCGAT GTAAGGAAGA CGCCGACCAA ATCCTAGAAA
 -775   GAAAGGAAGG ATAGGATCGG TTGATTGCTC CAGTGCAGGA TTTAGGACCT ATGGGATTAA GGTCTCGTAG GGGGCTGGTG
        CTTTCCTTCC TATCCTAGCC AACTAACGAG GTCACGTCCT AAATCCTGGA TACCCTAATT CCAGAGCATC CCCCGACCAC

                                                                                      XhoI
 -695   GCTGTAGTAC AAACTACGGA GAGAGAGGCC CTGGATTCCA TTCTCTAGAA CACACAAGCG CGCGCGCGCG CGCTCTCGAG
        CGACATCATG TTTGATGCCT CTCTCTCCGG GACCTAAGGT AAGAGATCTT GTGTGTTCGC GCGCGCGCGC GCGAGAGCTC
 -615   ATTAAAACCC TTTCAGAAAA TTTGATTACA AATCTAGAGA ATTTGTAGGG GTTAGGACTG GGAACAGAAT AGAAAGTGAG
        TAATTTTGGG AAAGTCTTTT AAACTAATGT TTAGATCTCT TAAACATCCC CAATCCTGAC CCTTGTCTTA TCTTTCACTC
 -535   ATTGAAAATC TTTCCCCAGG AGGAGGCAGA TGGTTTTGCA GGTAAAGACG CCTGGTGCCA AGCGTGGCTG CTTGAGTTCA
        TAACTTTTAG AAAGGGGTCC TCCTCCGTCT ACCAAAACGT CCATTTCTGC GGACCACGGT TCGCACCGAC GAACTCAAGT
 -455   GTCCCCGGAA CTCGGAAGGT GGAAGGTAAG ATCCTAGTCC TCCAAATTGT TCTCTAACCT CTACACCGCT CTGAGGCTCC
        CAGGGGCCTT GAGCCTTCCA CCTTCCATTC TAGGATCAGG AGGTTTAACA AGAGATTGGA GATGTGGCGA GACTCCGAGG
 -375   AGGGTACTTA CACACATACA CGGAAACAAT CAACCAGTCA AACAATAAAC GTAAAATATT ACAATTCTTT AGGGAATCCA
        TCCCATGAAT GTGTGTATGT GCCTTTGTTA GTTGGTCAGT TTGTTATTTG CATTTTATAA TGTTAAGAAA TCCCTTAGGT
 -295   AGGGTATTTG GTCTCTTAGG TAGGGGTCCA AAATCTCTGG GGATAGGGTT TAGAATCCTT TGGACAGGCT CATTACATCA
        TCCCATAAAC CAGAGAATCC ATCCCCAGGT TTTAGAGACC CCTATCCCAA ATCTTAGGAA ACCTGTCCGA GTAATGTAGT

                       SpeI
 -215   TGGGGGGCTT AAACGCACTA GTGGGGGAGG GGGACTGGGA ATCTTGGGAG GGCGGGTCAA ACCTTGGTGT CTGAGTAAGT
        ACCCCCCGAA TTTGCGTGAT CACCCCCTCC CCCTGACCCT TAGAACCCTC CCGCCCAGTT TGGAACCACA GACTCATTCA
 -135   CCTTCAAGGA CTGGTTAGCG CCCTTGAGGA CAGGGTTAGC AAAGAAATGC TGAAATTCTT TGGAGGTGAA TTTAGAGCAG
        GGAAGTTCCT GACCAATCGC GGGAACTCCT GTCCCAATCG TTTCTTTACG ACTTTAAGAA ACCTCCACTT AAATCTCGTC
                                                                   TSS
  -55   GAGACAAGAG AGCCAGAGTG GGAGTTAGGC TCTTAGAGGG CGGAGTCGGG GTATTTGGGG GCGGGGTTTG CATCACCCAG
        CTCTGTTCTC TCGGTCTCAC CCTCAATCCG AGAATCTCCC GCCTCAGCCC CATAAACCCC CGCCCCAAAC GTAGTGGGTC
  +26   GGTGCGGCTA AGGCGCCCAG ATGGCCAGTG AGCGCCACTG GGTTGCGGCT CCCAGCTTGC ACTCCCCGCG GCGGCTCTCA
        CCACGCCGAT TCCGCGGGTC TACCGGTCAC TCGCGGTGAC CCAACGCCGA GGGTCGAACG TGAGGGGCGC CGCCGAGAGT
 +106   GACGGGTGGG AGGGGACCAA AGTCGCCCGT GAAG
        CTGCCCACCC TCCCCTGGTT TCAGCGGGCA CTTC
```

Figure S2

```
Gli1_m  1     MFNPMTPPQVNSYSEPCCLRPLHSQGVPSMGTEGLSGLPFCHQANFMSGSQGYGAARETS  60
Gli1_h  1     MFNSMTPPPISSYGEPCCLRPLPSQGAPSVGTEGLSGPPFCHQANLMSGPHSYGPARETN  60
                          Y                                       Y

Gli1_m  61    SCTEGSLFPPPPPPRSSVKLTKKRALSISPLSDASLDLQTVIRTSPSSLVAFINSRCTSP  120
Gli1_h  61    SCTEGPLFSSP---RSAVKLTKKRALSISPLSDASLDLQTVIRTSPSSLVAFINSRCTSP  117

Gli1_m  121   GGSYGHLSIGTMSPSLGFPPQMSHQKGTSPPYGVQPCVPHDSTRGSMMLHPQARGPRATC  180
Gli1_h  118   GGSYGHLSIGTMSPSLGFPAQMNHQKGPSPSFGVQPCGPHDSARGGMIPHPQSRGPFPTC  177
                 Y                           y

Gli1_m  181   QLKSELDMMVGKCPEDPLEGDMSSPNSTGIQDHLLGMLDGREDLEREEKPEPESVYETDC  240
Gli1_h  178   QLKSELDMLVGKCREEPLEGDMSSPNSTGIQDPLLGMLDGREDLEREEKREPESVYETDC  237
                                                                     Y

Gli1_m  241   RWDGCSQEFDSQEQLVHHINSEHIHGERKEFVCHWGGCSRELRPFKAQYMLVVHMRRHTG  300
Gli1_h  238   RWDGCSQEFDSQEQLVHHINSEHIHGERKEFVCHWGGCSRELRPFKAQYMLVVHMRRHTG  297
Zn-Fingers                                                    Y

Gli1_m  301   EKPHKCTFEGCRKSYSRLENLKTHLRSHTGEKPYMCEQEGCSKAFSNASDRAKHQNRTHS  360
Gli1_h  298   EKPHKCTFEGCRKSYSRLENLKTHLRSHTGEKPYMCEHEGCSKAFSNASDRAKHQNRTHS  357
                            Y                  Y

Gli1_m  361   NEKPYVCKLPGCTKRYTDPSSLRKHVKTVHGPDAHVTKRHRGDGPLPRAQPLSTVEPKRE  420
Gli1_h  358   NEKPYVCKLPGCTKRYTDPSSLRKHVKTVHGPDAHVTKRHRGDGPLPRAPSISTVEPKRE  417
                  Y          Y

Gli1_m  421   REGGSGREESRLTVPESAM-PQQSPGAQSSCSSDHSPAGSAANTDSGVEMAGNAGGSTED  479
Gli1_h  418   REGGPIREESRLTVPEGAMKPQPSPGAQSSCSSDHSPAGSAANTDSGVEMTGNAGGSTED  477

Gli1_m  480   LSSLDEGPCVSATGLSTLRRLENLRLDQLHQLRPIGSRGLKLPSLTHAGAPVSRRLGPPV  539
Gli1_h  478   LSSLDEGPCIAGTGLSTLRRLENLRLDQLHQLRPIGTRGLKLPSLSHTGTTVSRRVGPPV  537

Gli1_m  540   SLDRRSSSSSSMSSAYTVSRRSSLASPFFPPGTPPENGASSLPGLTPAQHYMLRARYASAR  599
Gli1_h  538   SLERRSSSSSISSAYTVSRRSSLASPFFPPGSPPENGASSLPGLMPAQHYLLRARYASAR  597
                            Y                                  Y     Y

Gli1_m  600   GSGTPPTAAHSLDRMGGLSVPPWRSRTEYPGYNPNAGVTRRASDPARAADHPAPARVQRF  659
Gli1_h  598   GGGTSPTAASSLDRIGGLPMPPWRSRAEYPGYNPNAGVTRRASDPAQAADRPAPARVQRF  657
                                          Y  Y

Gli1_m  660   KSLGCVHTPPSVA-TGRNFDPHHPTSVYSPQPPSITENVAMDTRGLQEEPEVGTSVMGNG  718
Gli1_h  658   KSLGCVHTPPTVAGGGQNFDPYLPTSVYSPQPPSITENAAMDARGLQEEPEVGTSMVGSG  717
                                   y     Y

Gli1_m  719   LNPYMDFSSTDTLGYGGPEGTAAEPYEARGPGSLPLGPGPPTNYGPGHCAQQVSYPDPTP  778
Gli1_h  718   LNPYMDFPPTDTLGYGGPEGAAAEPYGARGPGSLPLGPGPPTNYGPNPCPQQASYPDPTQ  777
                 Y          Y          Y                 Y          Y

Gli1_m  779   ENWGEFPSHAGVYPSNKAPGAAYSQCPRLEHYGQVQVKPEQGCPVGSDSTGLAPCLNAHP  838
Gli1_h  778   ETWGEFPSHSGLYPGPKALGGTYSQCPRLEHYGQVQVKPEQGCPVGSDSTGLAPCLNAHP  837
                          Y         Y        Y

Gli1_m  839   SEGSPGPQPLFSHHPQLPQPQYPQSGPYPQPPHGYLSTEPRLGLNFNPSSSHSTGQLKAQ  898
Gli1_h  838   SEGPPHPQPLFSHYPQPSPPQYLQSGPYTQPPPDYLPSEPRPCLDFD-SPTHSTGQLKAQ  896
                           y       Y     Y      Y

Gli1_m  899   LVCNYVQSQQELLWEGRNRGGLPNQELPYQSPKFLGGSQVSQSPAKTPAAAAAAYGSGFA  958
Gli1_h  897   LVCNYVQSQQELLWEGGGREDAPAQEPSYQSPKFLGDSQVSPSRAKAP---VNTYGPGFG  953
                  Y                       Y                         Y

Gli1_m  959   PASANHKSGSYPAPSPCHETFTFVGVNRPSHRPAAPPRLLPPLSPCYGPLKVGDTNPSCGH  1018
Gli1_h  954   PNLPNHKSGSYPTPSPCHENFVVGANRASHRAAAPPRLLPPLPTCYGPLKVGGTNPSCGH  1013
                        Y                                  Y

Gli1_m  1019  PEVGRLGAGPALYPPPEGQVCNALDSLDLDNTQLDFVAILDEAQGLSPPLSHEQGDSSKN  1078
Gli1_h  1014  PEVGRLGGGPALYPPPEGQVCNPLDSLDLDNTQLDFVAILDEPQGLSPPPSHDQRGSSGH  1073
                          Y

Gli1_m  1079  TPSPSGPPNMAVGNMSVLLGSLPGETQFLNSSA  1111
Gli1_h  1074  TPPPSGPPNMAVGNMSVLLRSLPGETQFLNSSA  1106
```

Figure S3

A

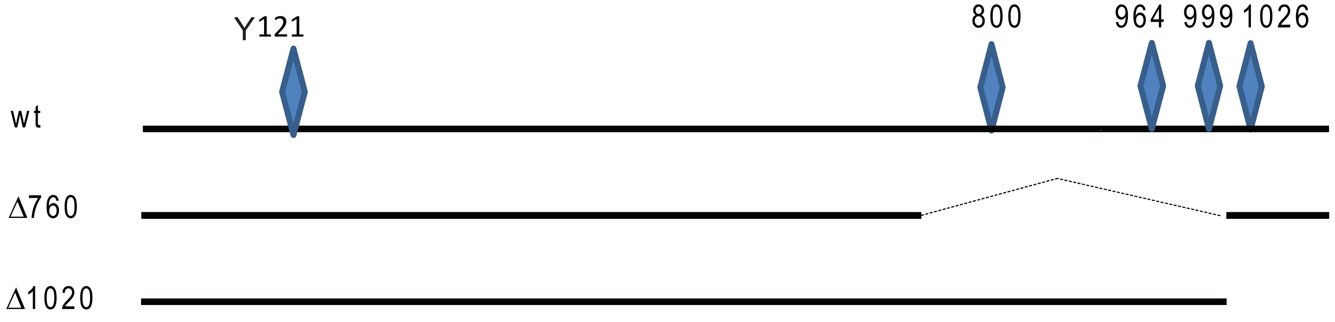

Y121
800
964
999
1026
wt
Δ760
Δ1020

B

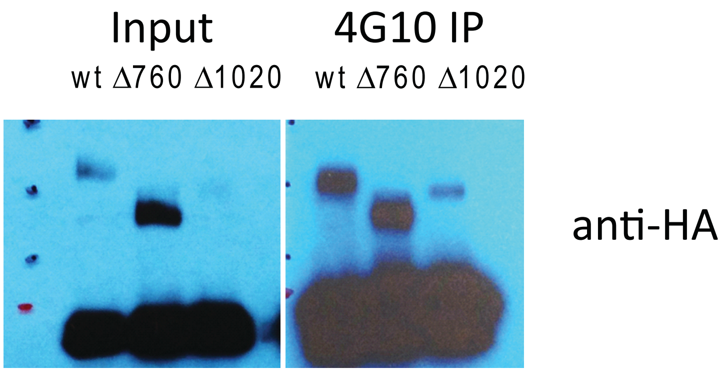

Input
4G10 IP
wt Δ760 Δ1020
wt Δ760 Δ1020
anti-HA

Figure S4

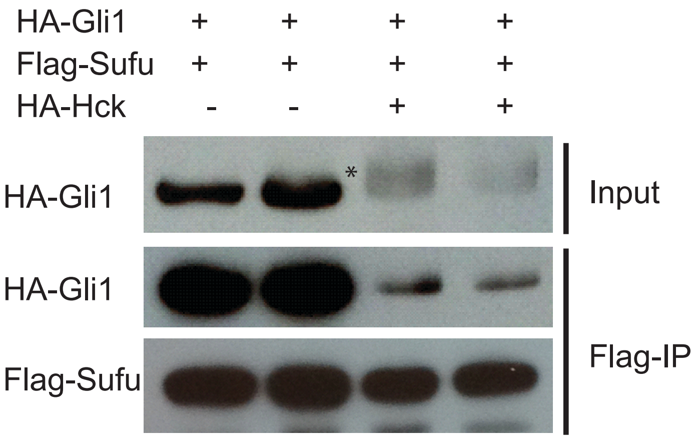

HA-Gli1 + + + +
Flag-Sufu + + + +
HA-Hck - - + +
HA-Gli1
*
Input
HA-Gli1
Flag-Sufu
Flag-IP

Figure S5 Hck level in human medulloblastoma and mouse medulloblastoma model

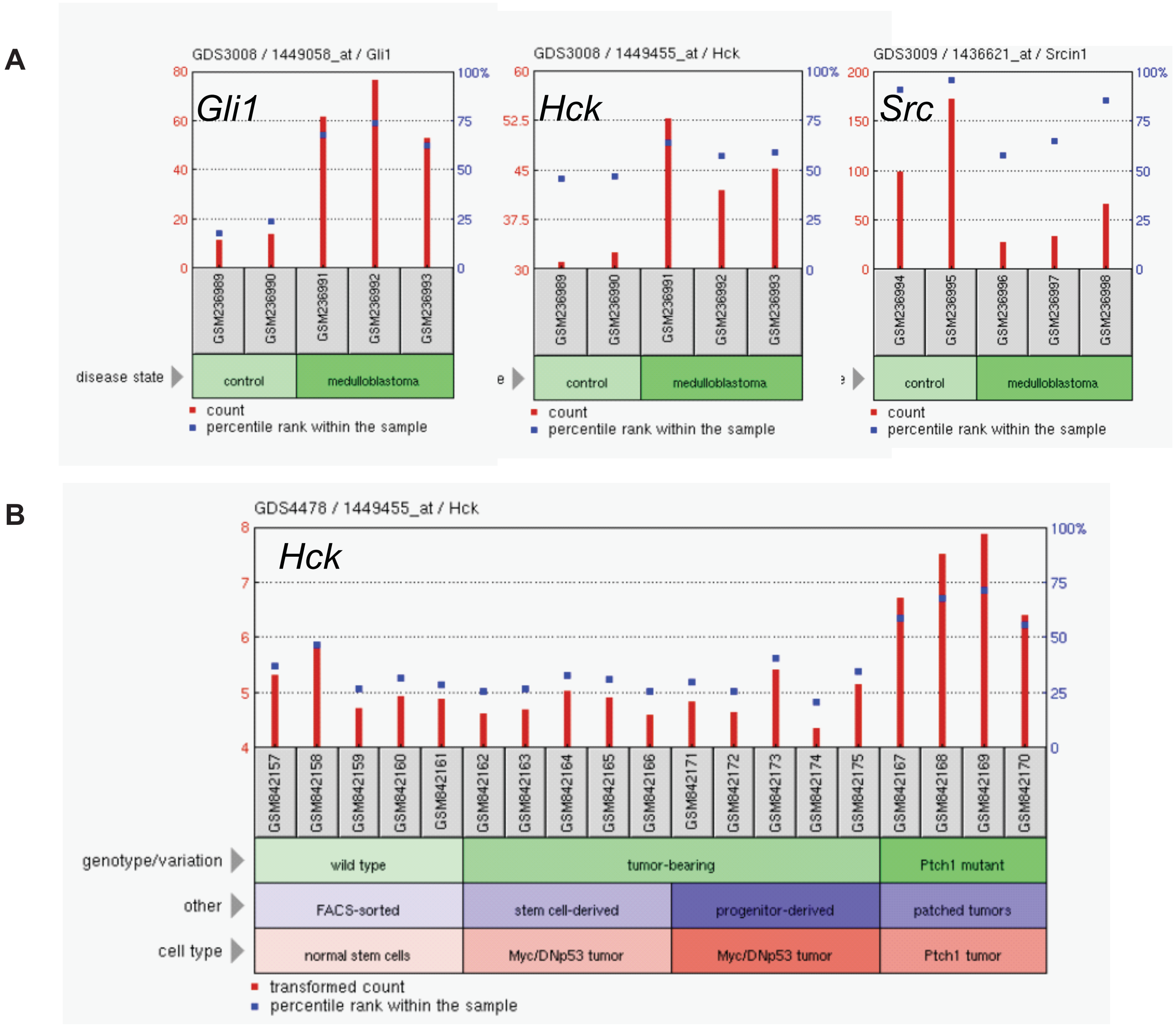

Supplement: Supplementary Information [file oncsis201538x1.pdf]
